# ATP redirects cytokine trafficking and promotes novel membrane TNF signaling *via* microvesicles

**DOI:** 10.1096/fj.201802386R

**Published:** 2019-02-18

**Authors:** Sanooj Soni, Kieran P. O’Dea, Ying Ying Tan, Kahori Cho, Eiko Abe, Rosalba Romano, Jiang Cui, Daqing Ma, Padmini Sarathchandra, Michael R. Wilson, Masao Takata

**Affiliations:** * Section of Anaesthetics, Pain Medicine, and Intensive Care, Faculty of Medicine, Chelsea and Westminster Hospital, Imperial College London, London, United Kingdom;; † Heart Science Centre, Harefield Hospital, National Heart and Lung Institute, Imperial College London, Harefield, United Kindom

**Keywords:** extracellular vesicles, protein signalling, cellular communication, danger signals

## Abstract

Cellular stress or injury induces release of endogenous danger signals such as ATP, which plays a central role in activating immune cells. ATP is essential for the release of nonclassically secreted cytokines such as IL-1β but, paradoxically, has been reported to inhibit the release of classically secreted cytokines such as TNF. Here, we reveal that ATP does switch off soluble TNF (17 kDa) release from LPS-treated macrophages, but rather than inhibiting the entire TNF secretion, ATP packages membrane TNF (26 kDa) within microvesicles (MVs). Secretion of membrane TNF within MVs bypasses the conventional endoplasmic reticulum– and Golgi transport–dependent pathway and is mediated by acid sphingomyelinase. These membrane TNF–carrying MVs are biologically more potent than soluble TNF *in vivo*, producing significant lung inflammation in mice. Thus, ATP critically alters TNF trafficking and secretion from macrophages, inducing novel unconventional membrane TNF signaling *via* MVs without direct cell-to-cell contact. These data have crucial implications for this key cytokine, particularly when therapeutically targeting TNF in acute inflammatory diseases.—Soni, S., O’Dea, K. P., Tan, Y. Y., Cho, K., Abe, E., Romano, R., Cui, J., Ma, D., Sarathchandra, P., Wilson, M. R., Takata, M. ATP redirects cytokine trafficking and promotes novel membrane TNF signaling *via* microvesicles.

Most cytokines are short lived and act locally in a paracrine or autocrine fashion, playing vital roles in disease pathogenesis (1). Despite a considerable body of literature detailing cytokine actions, our knowledge of cellular cytokine trafficking and release remains limited (2, 3). Activated macrophages synthesize proinflammatory cytokines in response to injurious or microbial challenges (1), which are then released *via* 2 distinct pathways depending on cytokine architecture: the classic or nonclassic secretory pathway. The majority of cytokines, including TNF, IL-6, and IL-12, are secreted *via* the classic pathway. This involves rapid transcription and translation of cytokines, which are conveyed from the endoplasmic reticulum (ER) to the Golgi complex. These proteins undergo sorting in the *trans*-Golgi network and are then transported to the cell surface *via* recycling endosomes (4–6). Several cytokines, such as the IL-1 family, are released *via* the poorly understood nonclassic pathway, which does not traffic through the ER and Golgi apparatus because of a lack of signal sequences (7).

Ongoing cellular stress during inflammatory states also results in the release of endogenous danger signals or damage-associated molecular patterns, which play a central role in activating and alerting immune cells to tissue distress (8). ATP is a ubiquitous nucleotide danger signal, vital for cell-to-cell communication. Although intracellular ATP concentration is around 3–10 mM, baseline extracellular ATP concentration is in the nanomolar range, allowing a 10^6^-fold gradient for ATP efflux. Transient increases in extracellular ATP are often seen in basic physiologic signaling, but larger increases, which are associated with cellular stress and injury, serve as a key danger signal in the inflammatory process (9). In this scenario, extracellular ATP binds to excitatory purinergic receptors on inflamed macrophages, promoting acute inflammation, and is essential for the release of nonclassically secreted proinflammatory cytokines, such as IL-1β and IL-18 (7, 10–12). Peculiarly, however, ATP has also been reported to inhibit the secretion of ER- and Golgi transport–dependant classically secreted cytokines from inflamed immune cells (13–17), in particular the very potent, proinflammatory cytokine TNF. This creates a major contradiction regarding the effects of danger signals on cytokine release. Although this may be a self-regulatory effect to limit excess development of inflammation, the cellular mechanisms, pathways, and, indeed, the rationale involved remain unexplained.

To address this long-standing paradox, here we carry out a systematic characterization of the effects of ATP on TNF production from LPS-stimulated macrophages, using combined *in vitro* and *in vivo* approaches. Our data demonstrate that ATP redirects TNF trafficking pathways, switching off soluble TNF (17 kDa) release from activated macrophages but instead preferentially packages transmembrane pro-TNF (26 kDa) within released microvesicles (MVs). We uncover a novel way of membrane TNF signaling *in vivo* and establish that these MVs are highly potent, inducing significant TNF-dependent inflammation and allowing long-range TNF signaling to target cells more effectively than soluble TNF. These data suggest mechanisms that potentially explain why previous anti-TNF strategies targeting soluble TNF have shown little benefit for acute inflammatory diseases such as sepsis or acute respiratory distress syndrome (ARDS) (18).

## MATERIALS AND METHODS

### Animal experimentation

All protocols were approved by the Ethical Review Board of Imperial College London, carried out under the authority of the UK Home Office in accordance with the Animals (Scientific Procedures) Act 1986, and reported in compliance with the *Animal Research: Reporting of* *In Vivo* *Experiments* guidelines (National Centre for the Replacement Refinement & Reduction of Animals in Research, London, United Kingdom). Seventy-six male C57BL/6 mice (Charles River, Wilmington, MA, USA) and 6 TNF^−/−^ mice (The Jackson Laboratory, Bar Harbor, ME, USA) aged between 7 and 8 wk [for bone marrow–derived macrophage (BMDM) harvesting] or 10–14 wk (for *in vivo* experimentation) were used. Mice were housed in individual ventilated cages (maximum number of 5/cage) and exposed to 12-h light/dark cycles. All experiments were initiated and completed during the light cycle, and no unexpected adverse effects were observed in any of the treatment groups.

### *In vitro* cell culture for MV production

RAW 264.7 macrophages (MilliporeSigma, Burlington, MA) (mycoplasma tested) were cultured in DMEM supplemented with 10% heat-inactivated fetal bovine serum and 1% penicillin-streptomycin-glutamine, at 37°C in a humidified 5% CO_2_ atmosphere. BMDMs were isolated and cultured as previously described by Manzanero (19). In brief, C57BL/6 mice were euthanized, and the trunks and legs of mice were sprayed with 70% ethanol solution. The femurs were isolated and flushed with 5 ml of sterile media to extract bone marrow cells. Bone marrow–containing medium was filtered, and cells were isolated (200 *g* 5 min at 4°C) and cultured for 5 d in 148-cm^2^ dishes in a humidified 5% CO_2_ atmosphere with medium supplemented with 50 ng/ml recombinant M-CSF (PeproTech, Rocky Hill, NJ, USA) to induce differentiation of bone marrow monocytes into macrophages (Supplemental Fig. S2*A*). Media were changed every second day. RAW cells and BMDMs were seeded at a density of 5 × 10^5^ cells per well for a 24-well plate or 2 × 10^6^ for a 10-cm dish and maintained in culture medium overnight prior to treatment.

To generate MVs with a proinflammatory phenotype, cells were pretreated with 1 μg/ml of Ultrapure LPS (*E. coli* O111:B4; InvivoGen, San Diego, CA, USA) for 1 h (20, 21). These inflamed macrophages were then stimulated with 3 mM ATP disodium salt (R&D Systems, Minneapolis, Minnesota, USA) or 2 U/ml recombinant sphingomyelinase (22) (MilliporeSigma) for either 15 min (BMDMs) or 1 h (RAW cells) to stimulate MVs (23). In some experiments, cells were pretreated with 10 μM batimastat (BB94) (24) [a broad-spectrum matrix metalloprotease inhibitor to inhibit TNF-α converting enzyme (TACE, also known as ADAM metallopeptidase domain 17) activity (British Biotech, Oxford, United Kingdom)], 25 µM desipramine (25) (R&D Systems) [to inhibit acid sphingomyelinase (ASM)] or 5 µg/ml brefeldin A (BFA) (26) (BioLegend, San Diego, CA, USA). Following stimulation, cell culture supernatant was harvested with 10 µl used for analysis of MV counts by flow cytometry. The remaining supernatant was treated with BB94 to prevent cleavage of MV-associated membrane TNF to soluble TNF, and then centrifuged at 200 *g* for 10 min at 4°C to remove any residual cells and large debris. Cell-free supernatant underwent high-speed centrifugation (20,000 *g* for 30 min at 4°C) to pellet MVs, which were then washed twice to remove contaminating factors (Supplemental Fig. S1*A*) (21). The MV pellet and MV-free supernatant were then analyzed with Western blotting and ELISA assay for target proteins.

### *In vivo* MV production and isolation

For the 2-hit LPS-ATP alveolar compartment treatment *in vivo*, randomly selected mice were anesthetized by intraperitoneal injection of ketamine (90 mg/kg) and xylazine (10 mg/kg). The vocal cords were identified using a microscope and an external light source, and a fine catheter was briefly passed 1 cm below the cords. Fifty nanograms Ultrapure LPS in 25 µl was instilled intratracheally as previously described (27, 28). The mice were suspended in an upright position for 30 s to allow equal bilateral distribution of the LPS. Animals were then placed in a heated box. After 30 min 25 µl of 5 mM ATP in saline, a physiologically relevant dose of ATP, which should produce bronchoalveolar lavage fluid (BALF) levels of ATP similar to those previously found in mouse models of acute lung injury (29), was instilled intratracheally while mice were still anesthetized. Thirty minutes after the second intratracheal challenge (length of treatment protocol 1 h), mice were euthanized with anesthetic overdose and exsanguination. An endotracheal tube was inserted *via* tracheostomy, and BALF was obtained by flushing and gently aspirating 700 μl of 0.9% saline in and out of the lungs *via* the endotracheal tube 3 times. This was kept on ice, and 10 µl was removed for MV count *via* flow cytometry. The remaining BALF was treated with BB94 and then processed as described above for MV pellet and MV-free supernatant analysis (Supplemental Fig. S3*A*).

### Cell analysis by flow cytometry

#### Cell surface staining in vitro

After collection of neat supernatant for MV harvesting, the remaining adherent cells were dissociated from each well using Versene solution (Thermo Fisher Scientific, Waltham, MA, USA) for 7 min at 37°C. Cell pellets were isolated by centrifugation and incubated with a fluorescence-conjugated antibody cocktail containing anti-CD45 (clone 30-F11; BioLegend), anti-CD11b (M1/70; BD Biosciences, San Jose, CA, USA), anti-TNF (MP6-XT22; BioLegend), and its corresponding isotype control, IgG1κ (MOPC-21; BioLegend), for 30 min at 4°C.

#### Intracellular staining in vitro

Cells were incubated with BD cytofix/cytoperm (BD Biosciences) at 37°C for 10 min to permeabilize and fix them. These cells were then washed twice with permeabilization wash buffer (0.2% saponin, 0.5% BSA, and 0.1% sodium azide in PBS), and antibody staining then followed as described above. RAW macrophages were identified as CD45^+^ and CD11b^+^, whereas BMDMs were identified as CD45^+^, CD11b^+^, and F4/80^-^positive (BM8; eBioscience, San Diego, CA, USA) (Supplemental Fig. S2*A*).

#### In vivo

Following doses of LPS and ATP, the lungs of mice were also removed and mechanically disrupted in BD cytofix/cytoperm using a GentleMacs dissociator (Miltenyi Biotec, Surrey, United Kingdom). Samples were then passed through 40-µm sieves, washed, and resuspended twice in permeabilization wash buffer to yield a fixed single cell suspension. Alveolar macrophages were stained with fluorescence-conjugated antibodies of anti-CD45, anti-CD11c (N418; BioLegend), anti-CD11b, anti-F4/80, and anti-TNF (Supplemental Fig. S3*B*).

All stained samples were analyzed using flow cytometry (CyAn ADP Analyzer; Beckman Coulter, Brea, CA, USA). Cell populations were analyzed in FlowJo software for their mean fluorescence intensity (geometric mean) for surface (unpermeabilized) or total (permeabilized) TNF expression.

### MV analysis by flow cytometry

In order to identify MVs using flow cytometry, 3 separate criteria had to be satisfied: *1*) size <1 µm, *2*) positive for specific precursor cell markers, and *3*) sensitivity to 0.1% Triton X-100 detergent (Supplemental Fig. 1*B*) (21). Ten microliters of culture supernatant was incubated with fluorescence-conjugated antibodies against CD11b and CD45 for RAW cell–derived and BMDM-derived MVs (Supplemental Figs. S1*B* and S2*B*). For *in vivo* alveolar macrophage–derived MVs, 10 μl BALF was incubated with fluorescence-conjugated antibodies against CD11c and CD45. Prior to flow cytometric analysis, stained samples were resuspended in 1 ml PBS and cotreated with a known quantity of AccuCheck 6 μm Counting Beads (Thermo Fisher Scientific) for the assessment of MV count. Forward scatter and side scatter (trigger threshold, 0.02) were used to elucidate a 1-µm gate that was delineated by specific sizing beads as previously described by Soni *et al*. (21).

### Intratracheal administration of MVs

BMDM-derived MVs were harvested from TNF^−/−^ mice and wild-type (WT) C57BL/6 mice and stimulated with LPS and ATP as described above (without BB94 treatment) to generate TNF-depleted and TNF-laden BMDM-derived MVs, respectively. MVs were counted using flow cytometry, and 4 × 10^6^ TNF-laden or TNF-depleted [doses of MVs that should produce similar BALF levels of MVs found in mouse models of acute lung injury (21)] were instilled intratracheally into the lungs of randomly selected recipient WT mice for 4 h by an investigator blinded to the treatment groups. At the end of the protocol, mice were euthanized, and BALF samples were taken. Using Qubit Assay (Thermo Fisher Scientific) and ELISA kits, BALF protein and chemokine (C-X-C motif) ligand 1 (CXCL1) levels (R&D Systems), respectively, were quantified in cell-depleted BALF.

Lungs were removed and mechanically disrupted to produce single cell suspensions, as previously described. Samples were then stained with antibodies at room temperature for 30 min to characterize each lung cell population: type 1 epithelial cells were identified as CD45^−^, CD31^−^ (MEC 13.3; BD Biosciences), epithelial cell adhesion molecule–negative (G8.8; BioLegend), and T1α^+^ (8.1.1; BioLegend) cells; type 2 epithelial cells as CD45^-^, CD31^−^, epithelial cell adhesion molecule–positive, and T1α positive (Supplemental Fig. S5*C*); lung neutrophils as CD45^+^, CD11b^+^, Ly6G+ (1A8; BioLegend), and LY6C+ (HK14; BioLegend); and lung monocytes as CD45^+^, CD11b^+^, LY6C^+^, and Ly6G^−^. Lung cell suspensions were also incubated with anti–intercellular adhesion molecule 1 (ICAM1) (YN1/1.7.4; eBioscience) and its corresponding isotype control, IgG2bκ isotype control (RTK4530; BioLegend), to ascertain epithelial cell ICAM1 expression.

### Cytokine analysis

Sandwich ELISAs (DuoSet ELISA; R&D Systems) were conducted to measure TNF content within cell- and MV-free supernatants and cell- and MV-free BALF as previously described by Soni *et al*. (21).

TNF analysis in MVs was performed *via* Western blotting (30, 31). In brief, MV pellets were treated with MV lysate buffer (0.5% Triton X-100 detergent and 1 mM PMSF in PBS) for 30 min on ice. Protein quantification was measured using Qubit assay, and the protein extracts (40 µg/sample) were heated, denatured, and loaded on a NuPAGE 4–12% Bis-Tris gel (Thermo Fisher Scientific). The samples subsequently underwent electrophoresis before being transferred to a nitrocellulose membrane. The membrane was treated with blocking solution (5% dry milk in Tris-buffered saline with 0.1% Tween-20) for 1 h and probed with one of the following primary antibodies: rabbit anti-TNF (1:1000) (3707s; Cell Signaling Technology, Danvers, MA, USA), goat anti-TACE (1:1000) (ab13535; Abcam, Cambridge, United Kingdom), and the loading control rabbit anti–β-actin (1:1000; Cell Signaling Technology). These were incubated in blocking solution overnight at 4°C, followed by horseradish peroxidase–conjugated secondary antibody for 1 h. The blots were visualized *via* luminol reagent (Santa Cruz Biotechnology, Santa Cruz, CA, USA) or SuperSignal West Femto Substrate (Thermo Fisher Scientific) for ECL detection, analyzed with GeneSnap (Syngene, Bangalore, India), and protein band intensity was expressed as ratio to β-actin.

### Confocal microscopy

Cells were seeded onto coverslips at 5 × 10^5^ and maintained in culture medium overnight. The following day, cells were treated as described above to produce MVs. Supernatants were taken; MVs were isolated as previously described and then placed on poly-l-ornithine–coated coverslips to encourage adherence for 6 h. Thereafter, both cells and MVs were washed with PBS 3 times, permeabilized with 0.5% Triton X-100, blocked with 3% bovine serum albumin for 30 min, and then incubated with 5 µg/ml goat polyclonal anti-TNF (AF 410; R&D Systems) or 5 µg/ml rabbit-polyclonal anti-CD11b (ab128797; Abcam) overnight in the dark at 4°C. Samples were washed and then incubated with donkey anti-goat (Alexa Fluor 594) for TNF or goat anti-rabbit (Alexa Fluor 488) for CD11b for 60 min. These were washed once again and incubated with DAPI intranuclear stain for 10 min. Coverslips were placed on slides with Mounting PermaFluor (Thermo Fisher Scientific) and viewed using an LSM880 nonlinear optical multiphoton confocal imaging system with an Axio Observer 1 microscope (Zeiss, Oberkochen, Germany). The objective lens used was a Plan Apochromat ×40/1.3 oil DIC UV VIS-IR (Zeiss). The imaging medium was oil, and the temperature was −20°C. The fluorochromes used were Alexa 488 and Alexa 594. .czi images were acquired using Zen software (Zeiss) and were then exported as 16-bit .tiff images. No image processing software was used.

### Electron microscopy

#### Transmission

Cells were stimulated in 35 × 15 mm plates to produce MVs as previously described. These were washed and fixed, and samples were incubated with 5 µg/ml anti-TNF antibody overnight. Samples were washed and incubated with goat anti-mouse 10 nm colloidal gold particles (BBI Solutions, Crumlin, United Kingdom). This was followed by further washing, incubation with 2.5% glutaraldehyde for 1 h, osmium tetroxide 1% for 1 h, then dehydrated in graded amounts of ethanol (25, 50, 70, 95, 100%; each for 20 min). Propylene oxide was added, and the dish was rotated until a layer appeared, which was aspirated and centrifuged. Araldite propylene was added at a ratio of 1:1, and samples were left on a rotator overnight. Samples were loaded into an embedding capsule, which was filled with araldite and polymerized in oven over 60°C for 48 h. Ultrathin sections (100 nm) were cut using a Diatome diamond knife (Diatome, Hatfield, PA, USA), floated onto distilled water, collected on grids, and stained with 2% uranyl acetate and lead citrate for 10 min in each solution. For the electron microscopy (EM) evaluation, we acquired images using the transmission electronic microscope Jeol 1200 EX (Tokyo, Japan) with a Gatan Bio Scan camera model (Pleasanton, CA, USA). DigitalMicrograph software (Gatan) was used to take 792 images, which were exported as JPEG files.

#### Scanning

Cells were seeded in 60 × 15–mm dishes and treated as previously described. Following treatment, the supernatant was removed, and glutaraldehyde 2.5% was added for 1 h. Samples were washed with sodium cocodylate buffer and incubated with osmium tetroxide (1%) for 1 h. The cells were once again washed, covered with tannic acid (1%) for 20 min, and dehydrated in graded amounts of ethanol (25, 50, 70, 95, 100%; each for 20 min). Samples were then dried using hexamethyldisilazane, fixed onto an aluminum stub, and viewed with the scanning electron microscope (Jeol JSM 6010 LA Analytical Scanning Electron Microscope).

### Small interfering RNA and cell transfection

RAW cells were grown in DMEM and were seeded into 6-well plates at 500,000 cells per well 16 h before experimentation. Cells were transfected with 1.5 µg ASM small interfering RNA (siRNA) or control siRNA (Qiagen, Hilden, Germany) according to the manufacturer’s instructions using HiPerfect Transfection Reagent (Qiagen). After 6 h, medium was changed, and cells were incubated for a further 24 h before treatment with either LPS or LPS and ATP.

### Statistical analysis

Shapiro-Wilk normality tests were carried out using IBM SPSS (IBM, Armonk, NY, USA). Comparisons between groups were performed by Student’s *t* tests or 1-way ANOVA (with Bonferroni correction or Tukey’s honestly significant difference test for multicomparison) for parametric data and by Wilcoxon rank sum and Mann-Whitney *U* tests or the Kruskal-Wallis test (with Dunn’s multiple comparison) for nonparametric data. All data were analyzed on Prism software (GraphPad Software, La Jolla, CA, USA), and are expressed as means ± sd or median, interquartile range (IQR), and minimum or maximum values, respectively. A value of *P* < 0.05 was defined as the minimum threshold for statistical significance.

## RESULTS

### ATP inhibits soluble TNF secretion

**Figure 1:**
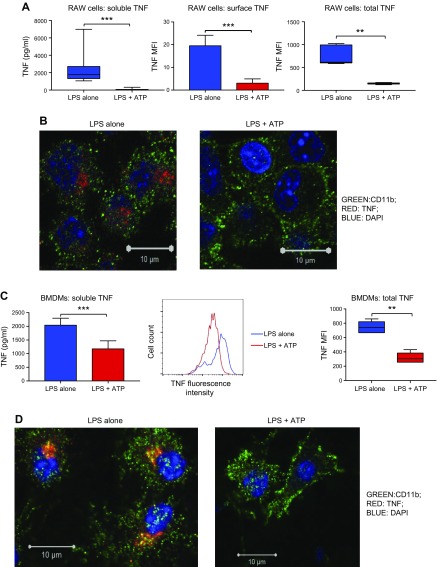
ATP inhibits soluble TNF release. *A*) RAW 264.7 macrophages exposed either LPS alone (1 µg/ml, 2 h) or 2-hit injury model with LPS (1 µg/ml, 1 h) followed by ATP (3 mM, 1 h). LPS induced soluble TNF secretion into cell- and MV-depleted supernatants, which was almost entirely abolished by ATP treatment (left, *n* = 9–10). ATP reduced both surface [middle, represented as mean fluorescence intensity (MFI), *n* = 5–6] and total cellular TNF expression (right, *n* = 5–6). *B*) Confocal images clearly showed disappearance, rather than accumulation, of cellular TNF from the RAW macrophages upon ATP exposure (*n* = 3). *C*) To confirm these results in primary cells, we exposed mouse BMDMs to LPS (1 µg/ml, 1 h) followed by ATP (3 mM, 15 min). Similarly, ATP inhibited soluble TNF secretion (left, *n* = 6) and reduced total cellular TNF expression (middle and right, *n* = 6). *D*) Confocal images of BMDMs illustrating the reduction of cellular TNF expression following ATP treatment (*n* = 3). Cells were also checked for TNF expression under nonstimulated conditions (*i.e.*, in PBS or medium) and, as expected, TNF was not detected in this sterile or noninflamed environment (Supplemental Fig. S1*G*). Parametric or nonparametric data are displayed as means ± sd or box-whisker plots showing the median, IQR, and minimum or maximum values, respectively. Scale bars, 10 µm. **P < 0.01, ***P < 0.001.

To fully characterize the effect of ATP on TNF release, we adopted *in vitro* 2-hit injury models using RAW 264.7 macrophages and mouse primary BMDMs (Supplemental Fig. S1*A*), in which macrophages were exposed to a primary insult with LPS followed by a second hit with ATP. As previously reported (13–17), ATP treatment of LPS-primed macrophages significantly inhibited soluble TNF secretion into culture supernatants (depleted of cells and MVs) (**Fig. 1*A, C***). Yet, rather than accumulating within cells, both surface and total cellular TNF content, as assessed by flow cytometry (Fig. 1*A, C*) and confocal microscopy (Fig. 1*B, D*, and Supplemental Fig. S1*D*, *E*), markedly decreased following ATP, indicating that TNF disappeared rapidly during this short period of ATP stimulation. Thus, we explored the whereabouts of this missing TNF.

### ATP preferentially packages membrane TNF within MVs

**Figure 2:**
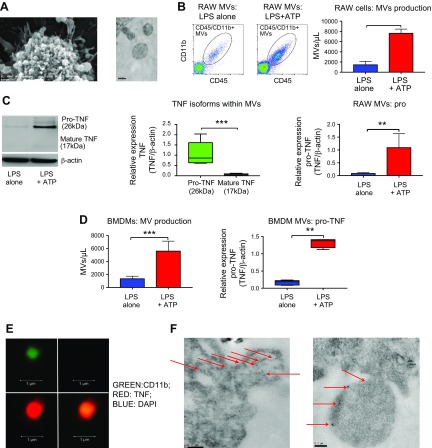
ATP preferentially packages membrane TNF within shed MVs. *A*) Scanning (left; scale bar, 1 µm) and transmission (right; scale bar, 0.2 µm) EM of RAW cells illustrates membrane blebbing and MV formation in response to ATP. *B*) RAW cell–derived MVs, identified as CD45^+^ or CD11b^+^ particles by flow cytometry (left and middle), significantly increased in response to ATP (right; *n* = 6–10). *C*) These MVs contained TNF, accounting for the above missing TNF from the cells but in the form of 26 kDa transmembrane pro-TNF isoform rather than 17 kDa soluble mature TNF (left and middle; *n* = 4–8). ATP packaged substantial amounts of pro-TNF within MVs (right; *n* = 4–8). *D*) To confirm these results in primary cells, we exposed mouse BMDMs to LPS (1 µg/ml, 1 h) followed by ATP (3 mM, 15 min). Similarly, ATP induced significant release of MVs (left; *n* = 6), which contained substantive amounts of pro-TNF (right; *n* = 6). *E*) Confocal microscopy illustrates that these BMDM-derived MVs, identified as CD11b^+^ (green, top left) particles negative for nuclear materials (DAPI^−^, top right), actually contained TNF (red, bottom left, colocalization shown in the bottom right combined image). Scale bars, 1 µm. *F*) Immune EM demonstrates transfer of pro-TNF (black dots, red arrows) from BMDMs to MVs during their formation, and these pro-TNF molecules gradually localized to MV membrane surface (hence, membrane TNF) (right). Scale bars, 0.2 µm. Parametric or nonparametric data are displayed as means ± sd or box-whisker plots showing the median, IQR, and minimum or maximum values, respectively. **P < 0.01, ***P < 0.001.

Accumulation of extracellular ATP after tissue stress also causes blebbing of cellular membranes and subsequent MV formation (23, 32). We confirmed that ATP actually induces MV formation and release from macrophages in our models using EM and flow cytometry (**Fig. 2*A, B, D***, and Supplemental Figs. S1*B*, *F*, and 2*B*). MVs generated in response to purinergic stimuli have been shown to work as an important vehicle for the release of nonclassically secreted cytokines such as IL-1β (33–37). As the presence of TNF within MVs has been previously suggested (21, 38), we hypothesized that these macrophage-derived MVs could also harbor TNF, thereby accounting for the missing TNF. Using Western blotting, we found that these MVs indeed contained a significant amount of TNF, and, very intriguingly, in the isoform of transmembrane pro-TNF (26 kDa) rather than the normally secreted, soluble (17 kDa) mature isoform (Fig. 2*C, D*). Confocal microscopy confirmed the accumulation of TNF within these MVs (Fig. 2*E*), and immune EM indicated that macrophages actively package TNF within MVs during the process of their blebbing and formation, which eventually localizes to the vesicle membrane (Fig. 2*F*). These data comprehensively demonstrate that ATP preferentially packages the membrane TNF isoform within MVs while switching off soluble TNF release.

### *In vivo*, ATP packages membrane TNF within alveolar macrophage–derived MVs

To investigate whether this phenomenon is replicated *in vivo*, we chose to examine the pulmonary alveolar space, which offers a semiclosed body compartment not exposed to rapid dilution and wash-out effects of blood flow and is thus ideal for studying MV signaling *in vivo*. The alveolar space also allows us to directly access and harvest alveolar macrophages, which are the primary source of TNF within this space (39). Furthermore, we hoped to clarify the precise profile of TNF release within the alveoli because TNF has been shown to play a crucial role in the pathophysiology of inflammatory lung diseases, such as acute lung injury and ARDS (27, 40).

**Figure 3:**
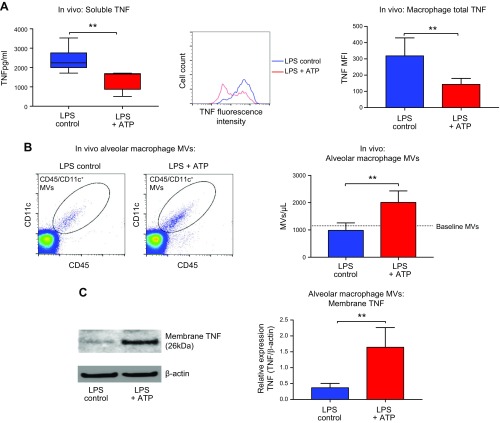
*In vivo*, ATP selectively packages membrane within alveolar macrophage–derived MVs while simultaneously switching off soluble TNF release. *A*) Mice were exposed to an *in vivo* 2-hit injury model: intratracheal LPS (50 ng, in 25 µl saline) instilled into murine lungs for 30 min followed by 5 mM ATP (in saline, 25 µl) for 30 min. LPS control mice consisted of intratracheal LPS followed by intratracheal saline. LPS induced soluble TNF secretion into alveoli (measured in cell- or MV-depleted BALF samples), which was substantively decreased by ATP (left; *n* = 5–6). ATP also caused a reduction in total cellular TNF content (measured by flow cytometry) in LPS-stimulated alveolar macrophages (middle and right), confirming that TNF was not internalized or did not accumulate within cells but disappeared from the cells (*n* = 5). *B*) In addition to inhibiting soluble TNF release, ATP significantly stimulated the release of MVs from alveolar macrophages, identified as CD45^+^CD11c^+^ particles by flow cytometry (*n* = 5). *C*) As *in vitro*, *in vivo*–derived MVs contained almost exclusively membrane TNF. Consequently, *in vivo* ATP preferentially packaged membrane TNF within MVs compared with LPS control MVs (*n* = 4). Parametric and nonparametric data are displayed as means ± sd or box-whisker plots showing the median, IQR, and minimum or maximum values, respectively. **P < 0.01.

We developed a 2-hit intra-alveolar inflammation model in mice (Supplemental Fig. S3*A*): intratracheal instillation of low-dose LPS induced activation of alveolar macrophages, leading to the release of soluble TNF into BALF (**Fig. 3*A***) without inducing the release of MVs (Fig. 3*B*). When ATP was instilled following LPS, it significantly reduced soluble TNF secretion from macrophages while diminishing cellular TNF content (Fig. 3*A*), as well as inducing a significant increase in MV production (Fig. 3*B* and Supplemental Fig. S3*C*). As *in vitro*, these *in vivo–*generated MVs contained a substantial amount of membrane TNF (Fig. 3*C*), indicating that ATP also switches off soluble TNF release and packages membrane TNF within MVs *in vivo*.

### Packaging of TNF within MVs is mediated *via* an unconventional Golgi-independent pathway

**Figure 4:**
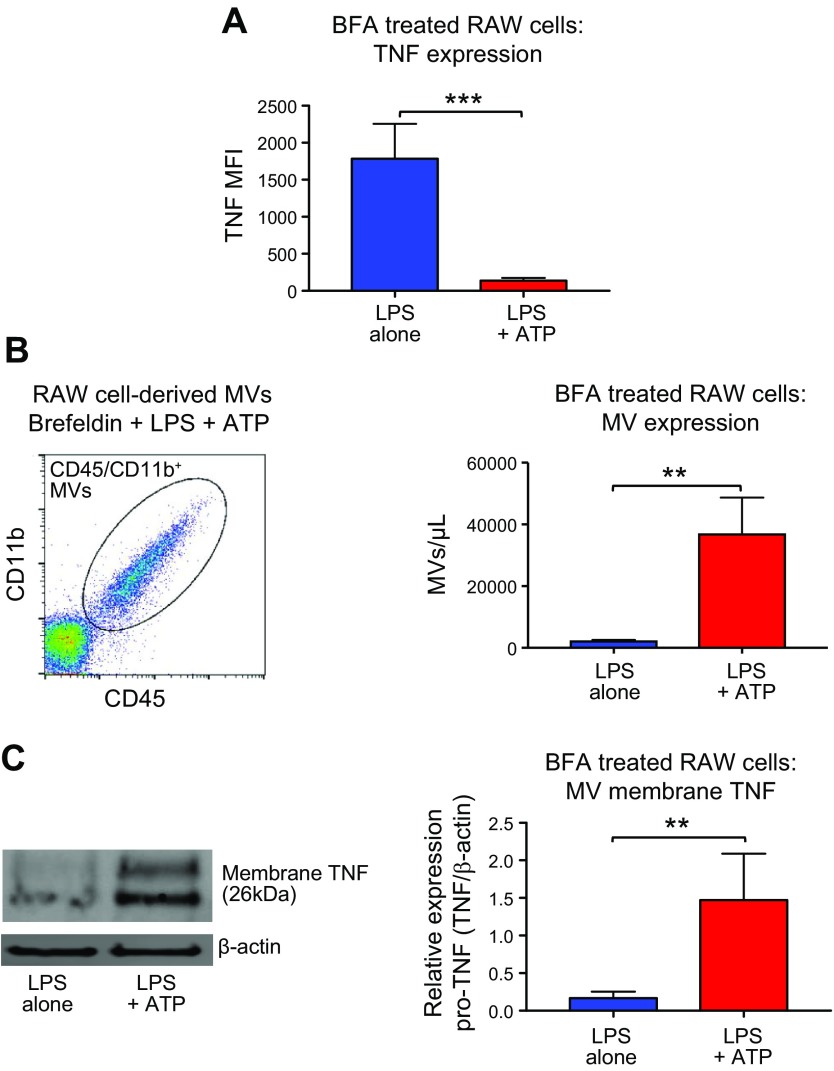
ATP secretes membrane TNF within MVs *via* an unconventional, ER- and Golgi-independent pathway. *A*) RAW macrophages were pretreated with BFA (5 µg/ml), an inhibitor of classic cytokine secretory pathway, in order to block TNF secretion in our *in vitro* 2-hit model. As expected, BFA completely abolished LPS-induced, soluble-TNF release. However, even in the presence of BFA (which blocked ER- and Golgi-dependent protein transport), ATP caused a dramatic reduction in total cellular TNF content, demonstrating that TNF is still secreted in some way. TNF expression in BFA-treated cells incubated in PBS was undetectable, confirming that the accumulation of TNF within macrophages was because of LPS rather than an effect of BFA itself. *B*) MVs released from BFA-treated RAW cells were identified *via* flow cytometry as CD45^+^ and CD11b^+^ (left), and ATP caused a significant increase in MV production in these cells (right; *n* = 4). *C*) These MVs contained a substantial amount of membrane TNF (left and right; *n* = 4). Data are displayed as means ± sd. **P < 0.01, ***P < 0.001.

To explore the mechanisms behind this phenomenon, experiments were carried out in the presence of BFA, an inhibitor of ER- and Golgi-dependent protein transport that is vital for classic secretion of cytokines (26, 41–43). It is well established that BFA prevents the release of soluble TNF (26), leading to intracellular accumulation of TNF (42, 43), but does not inhibit the release of nonclassically secreted proteins such as IL-1β (12). As expected, we found that the presence of BFA completely abrogated soluble TNF release from LPS-stimulated macrophages into culture supernatants (undetectable), and, surprisingly, macrophage cellular TNF content was not increased but, on the contrary, dramatically reduced in response to ATP (**Fig. 4*A***), indicating that TNF is still secreted. ATP caused a significant release of MVs from BFA-treated macrophages (Fig. 4*B*), and these MVs contained a substantial amount of membrane TNF (Fig. 4*C*). Thus, ATP redirects intracellular TNF trafficking from the classic ER- and Golgi-dependent pathway to an unconventional, ER- and Golgi-independent route, secreting TNF within MVs in a similar fashion to IL-1β.

### ASM switches TNF secretion to a nonclassic pathway

**Figure 5:**
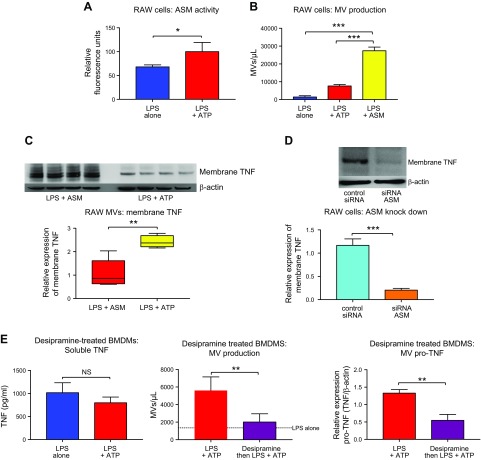
Switch of TNF secretion to nonclassic pathway is mediated by ASM. *A*) ASM activity was increased in RAW macrophages following ATP treatment (*n* = 3). *B*) LPS-primed RAW cells were treated with recombinant sphingomyelinase, which caused a marked increase in MV release compared with LPS alone or LPS- or ATP-treated RAW cells (*n* = 4–9). *C*) These ASM-generated MVs contain substantial amounts of membrane TNF compared with ATP-generated MVs (*n* = 4–7). *D*) Knockdown of ASM in RAW macrophages using siRNA prevented packaging of membrane TNF within MVs (*n* = 4). *E*) To confirm these results in primary cells, BMDMs were pretreated with the ASM inhibitor desipramine (25 mM) prior to exposure to LPS or LPS and ATP. Desipramine prevented ATP-induced reduction in soluble TNF release (left; *n* = 4), markedly attenuated ATP-induced MV release (middle; *n* = 4), and significantly reduced packaging of membrane TNF within MVs (right; *n* = 4–5). Parametric and nonparametric data are displayed as means ± sd or box-whisker plots showing the median, IQR, and minimum or maximum values respectively. NS, not significant. *P < 0.05, **P < 0.01, ***P < 0.001.

ASM has been shown to play a key role in nonclassic secretion of cytokines from cells (22). We hypothesized that ASM is responsible for this switch from classic to unconventional nonclassic TNF secretion and addressed this hypothesis in our *in vitro* 2-hit model. First, we found that ASM activity of RAW macrophages was increased after ATP treatment (**Fig. 5*A***). Next, when LPS-primed macrophages were exposed to bacterial sphingomyelinase, there was a substantive increase in MV release, even greater than after ATP treatment (Fig. 5*B* and Supplemental Fig. S4*A*), associated with reductions in soluble TNF release and cellular TNF content (Supplemental Fig. S4*B*, *C*). These ASM-generated MVs contained considerably more membrane TNF than ATP-generated MVs (Fig. 5*C*). To directly prove ASM involvement, we knocked down ASM in RAW macrophages using siRNA and then exposed these macrophages to our 2-hit model. We found that membrane TNF secretion *via* MVs in response to ATP was almost completely abolished where ASM had been silenced (Fig. 5*D*). To further confirm these results in primary cells, we pretreated BMDMs with the ASM inhibitor desipramine prior to exposure to LPS and ATP. Desipramine prevented the ATP-induced inhibition of soluble TNF release, attenuated ATP-induced MV release, and significantly inhibited packaging of membrane TNF within MVs (Fig. 5*E*). These data clearly demonstrate a critical role for ASM in this novel ATP-induced membrane TNF secretion *via* MVs.

### Membrane TNF is stable within MVs

**Figure 6:**
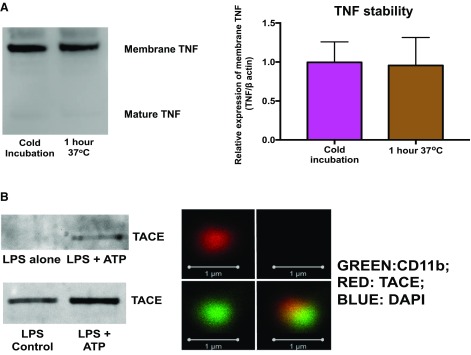
Membrane TNF is stable within macrophage-derived MVs. *A*) Membrane TNF is harbored in a stable, protected environment within MVs and was not spontaneously cleaved into the mature 17 kDa isoform despite being incubated at 37°C for 1 h. (*n* = 4). *B*) ATP also packages TACE into RAW cell–derived MVs (top) and *in vivo*–derived MVs (bottom). Confocal images of BMDM-derived MVs demonstrate the presence of TACE within BMDM-derived MVs. Scale bars, 1 μm. Data are displayed as means ± sd.

During conventional TNF trafficking, pro-TNF (26 kDa) within the cells is transported to the cell surface (termed membrane TNF), where it can undergo cleavage into soluble TNF (17 kDa) (2, 3) once TACE is activated (44, 45). Because membrane TNF may potentially be cleaved into soluble TNF or degraded by various proteases in these vesicles, we investigated the stability of membrane TNF within the MVs. RAW macrophage–derived MVs were washed and centrifuged without the addition of matrix metalloproteinase inhibitor BB94 (our standard procedure is to add BB94 to avoid any artifactual breakdown of membrane TNF by matalloproteinases including TACE; see Supplemental Fig. S1*A*) and incubated at 37°C for 1 h. These MVs were then compared with samples that were processed in the normal fashion (treated with BB94) and then kept on ice for 1 h. Surprisingly, there was no difference in membrane TNF content and no increase in soluble TNF in the supernatant (**Fig. 6*A***), despite the fact that TACE was expressed in these MVs as well as in BMDM- or *in vivo–*derived MVs (Fig. 6*B*). Although it is possible that TACE may interact with membrane TNF in response to further stimuli, particularly when MVs are approaching their target cells, our data showed that membrane TNF packaged within these vesicles is intrinsically very stable.

### MVs are a novel vehicle for membrane TNF signaling

**Figure 7:**
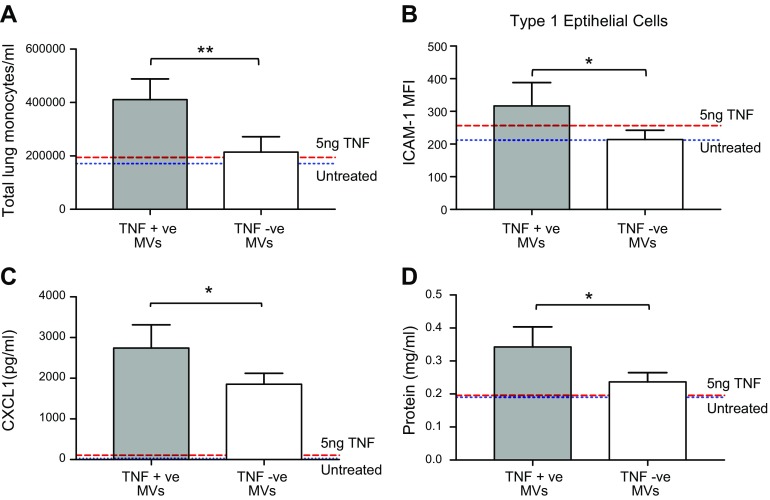
MVs provide a vehicle for membrane TNF signaling, causing significant inflammation *in vivo*. Membrane TNF containing BMDM-MVs from WT mice (TNF^+^ve MVs) were instilled intratracheally into the lungs of mice. Their biologic effects were compared with intratracheal instillation of BMDM-MVs taken from TNF^−/−^mice (TNF^−^ve MVs) and intratracheal high-dose recombinant (*i.e.*, soluble) TNF (50 µl 100 ng/ml). TNF^+^ve MVs caused significant increases in infiltrating inflammatory monocytes in lung tissue (flow cytometry) (*A*), ICAM1 expression on alveolar epithelial cells (flow cytometry) (*B*), BALF CXCL1 levels (by ELISA) (*C*), and BALF protein (by Qubit assay) (*D*) compared with TNF−ve MVs and intratracheal soluble TNF. Data are displayed as means ± sd (*n* = 4). *P < 0.05, **P < 0.01.

Finally, we assessed whether the membrane TNF within macrophage-derived MVs is biologically active *in vivo*. We generated MVs from BMDMs (with LPS followed by ATP) that were harvested from either WT or TNF^−/−^ mice. These MVs were instilled intratracheally into the lungs of WT mice with their biologic effects compared. BMDM-derived MVs from WT mice (containing membrane TNF) caused significant inflammation *in vivo* compared with MVs from TNF-deficient mice as measured across 4 different modalities: infiltrating inflammatory cells (monocytes), inflammation in alveolar epithelial cells (ICAM1 expression), proinflammatory cytokine release from target cells (BALF CXCL1), and lung-permeability change (BALF protein) (**Fig. 7** and Supplemental Fig. S5*B*, *D*).

To test the signaling capability of membrane TNF within MVs in comparison with soluble TNF, a large dose of recombinant TNF was also instilled intratracheally into the lungs of WT mice. This dose far exceeded the amount of membrane TNF within these MVs and would have produced a much higher TNF concentration within BALF than that found in mouse models of acute lung injury (46, 47). However, unlike the membrane TNF within MVs, this dose of soluble TNF did not cause significant inflammation (Fig. 7), indicating that the form in which TNF is secreted has a crucial impact on its *in vivo* effect. This suggests that soluble TNF is quickly degraded or neutralized in body fluid by inhibitors such as TNF receptors (48), whereas membrane TNF enclosed within MVs can be better protected, allowing more efficient signaling over a longer distance, thereby causing significant intra-alveolar inflammation *in vivo*.

## DISCUSSION

This study provides several novel insights into cytokine trafficking and TNF signaling. It is widely regarded that cytokines are released *via* one of the 2 distinct intracellular pathways (*i.e.*, the classic or nonclassic secretory pathway). Our data dismiss this long-held dogma and demonstrate that TNF, the best-studied and classically secreted cytokine, can be released in an unconventional, nonclassic, ER- and Golgi-independent manner. Rather than being anti-inflammatory and suppressing the entire TNF secretion, danger signals such as ATP can totally redirect TNF trafficking, inhibiting soluble TNF release and packaging the transmembrane pro-TNF isoform into potent, proinflammatory MVs. By directly comparing the inflammatory effects of TNF carried by MVs *vs.* its soluble counterpart, we have demonstrated that MVs uniquely protect enclosed TNF, allowing effective and potentially long-range TNF signaling to target cells *in vivo*. Furthermore, our study has uncovered an unconventional, novel third way in which TNF signals *in vivo*: although membrane TNF has been thought to signal only *via* direct cell-to-cell contact, membrane TNF can be secreted within shed vesicles, subsequently activating target cells without cell contact *per se*.

During inflammation, cells are exposed to a primary insult, such as a bacterial infection, hypoxia, or autoimmune reaction. Continued cellular stress subsequently results in release of danger signals from damaged cells, which accentuates inflammation by activating adjacent immune cells. ATP is a typical endogenous danger signal originating from large cytosolic stores and released into the extracellular space once cell membrane integrity is lost. These consecutive insults (2 hits) are required for macrophages to release nonclassically secreted cytokines, such as IL-1β and IL-18 (49). Because ATP is also a potent stimulator of MV release (23, 32), MVs have been implicated as specialist vehicles carrying these nonclassic proteins (33, 34). In contrast, classically secreted cytokines, such as TNF, have well-defined ER- and Golgi-dependent trafficking pathways that are totally different from the nonclassically secreted cytokines. Surprisingly, it has been reported that ATP paradoxically reduces TNF secretion from inflamed cells and consequently has anti-inflammatory affects (13–17). However, rather than being anti-inflammatory and suppressing entire TNF secretion, our results provided clear evidence, for the first time, that ATP indeed preferentially packages TNF within MVs in a fashion similar to IL-1β and that TNF loaded in these MVs induces significant inflammation *in vivo*.

Using pharmacological inhibitors and gene-silencing techniques, we performed detailed mechanistic studies to address the cellular pathways involved in this switch in TNF release. BFA is a well-established inhibitor of ER- and Golgi-dependent protein transport that abolishes classic cytokine secretion (26, 41–43) but does not inhibit the release of nonclassic cytokines (12). Utilizing BFA, we demonstrated that ATP redirects intracellular TNF trafficking from the classic ER- and Golgi-dependent pathway to an unconventional ER- and Golgi-independent route, secreting TNF within MVs. Furthermore, we identified ASM as a potential contender for the downstream mechanism for this ATP-induced switch because it has a key role in nonclassic cytokine secretion from cells, particularly IL-1β (22). Consistent with this hypothesis, ATP activated ASM in our models, whereas recombinant bacterial sphingomyelinase, like ATP, inhibited soluble TNF production and packaged membrane TNF within MVs. When cells were preincubated with desipramine, an inhibitor of ASM (50), and in cells where ASM had been genetically silenced, we found that membrane TNF secretion *via* MVs in response to ATP was almost completely abolished, establishing a critical role for ASM in this process.

Soluble TNF (17 kDa protein) and its precursor form membrane TNF (26 kDa protein) are both biologically active (51), but the latter has been thought to signal only *via* direct cell-to-cell contact (52). Our data demonstrate an entirely novel way in which membrane TNF signals *in vivo* in which the transmembrane isoform of TNF can be released within shed vesicles. These membrane TNF molecules, carried within MVs in a stable manner (*i.e.*, not cleaved into soluble TNF or degraded for a significant period of time), subsequently activate distant target cells without cell-to-cell contact *per se*. Using MVs from TNF knockout mice, we provided firm evidence that membrane TNF within MVs is biologically potent, initiating considerable inflammation within the mouse lungs *in vivo*.

By taking advantage of the alveolar space as a semiclosed body compartment, we directly compared the signaling efficacy of TNF carried by MVs against soluble TNF in our mouse lung inflammation model. Our results indicate that membrane TNF within MVs is more biologically potent than soluble TNF at equal or even higher doses. This strongly suggests that soluble TNF is quickly degraded or neutralized in body fluid (alveolar lining fluid) by inhibitors such as TNF receptors (48), whereas membrane TNF enclosed within MVs can be better protected, allowing more efficient signaling over a longer distance, thereby causing significant intra-alveolar inflammation *in vivo*. Interestingly, this may explain why previous studies instilling soluble or recombinant TNF into the lungs of mice have been unable to replicate lung injury (53) despite a substantial body of evidence for TNF involvement in the pathogenesis of acute lung injury. Furthermore, a fundamental difference in signaling mechanisms may exist when TNF is delivered in the form of membrane TNF within MVs rather than soluble TNF. For example, soluble TNF forms noncovalent trimers that tend to dissociate into nonfunctional monomers (54), whereas membrane TNF is more stable and fully functional once expressed on the cell surface. Hence, MVs expressing the membrane TNF isoform may be able to produce inflammation more efficiently than soluble TNF.

Our data indicating the significant difference in signaling efficiency between MV-enclosed TNF *vs.* soluble TNF may also offer important, previously unappreciated clinical implications. Clinical trials of anti-TNF therapy have shown little beneficial impact in acute inflammatory diseases such as sepsis or ARDS despite ample preclinical evidence of TNF involvement in their pathophysiology (18). In severe sepsis and ARDS, substantive cell injuries and damage-associated molecular pattern release take place, which, according to our data, would lead to secretion of TNF in the MV-enclosed, rather than soluble, form. Standard intravenous antibody–based blocking treatments may not be very effective to inhibit the effects of TNF conveyed by MVs, in particular within the alveolar space where TNF released from the captured MVs exerts its effects on target epithelial cells in an extreme spatial proximity. Our study suggests that targeting MV-mediated TNF signaling, by means of reducing MV uptake or production, may be essential to block all of the TNF biologic effects in acute inflammatory diseases such as ARDS, which warrants further investigation.

There are some limitations to our work. First, cells also release other species of extracellular vesicles, including exosomes in response to ATP, albeit to a lesser extent than MVs in acute phases (55). We did not explore the capacity of exosomes to carry TNF, but this may be important because exosomes have also been shown to harbor cytokines (20, 56). Although our centrifugation protocols (20,000 *g* for 30 min) were designed to remove cells and debris without introducing exosomes into the MV pellet [exosomes would not normally pellet at speed <100,000 *g* (23, 57)], there is still a potential for contamination, albeit unlikely, particularly because all vesicles seen in our transmission electronic microscope photos were >100 nm in size. Second, we used a CyAn ADP flow cytometer, which has previously been used in the literature to characterize MVs (58) but is designed primarily for cell analysis. Flow cytometry of newer generations with better sensitivity and resolution could help in improving efficiency and precision of our MV analysis. However, there are still no flow cytometers that are able to reliably measure all subpopulations of MVs because of current technical limitations. We used our established, rather conservative experimental approach to detect relevant MVs using criteria involving particle size, surface marker expression, and detergent sensitivity (21), and we believe that this methodology, although missing some MVs of smaller sizes, is appropriate for the analyses in this study, for which reproducibility of MV identification and detection rather than the absolute numbers is crucial. Third, we did not explore whether the phenomenon presented here is exclusive for TNF or whether it also occurs for other classically released cytokines such as IL-6 and IL-12, which warrants further investigation. Fourth, although we have concentrated upon the proinflammatory properties of macrophage MVs as vehicles for membrane TNF signaling, MVs also can carry some amount of anti-inflammatory cargo (59). Whether macrophage-derived MVs have an overall proinflammatory *vs.* anti-inflammatory nature likely depends on the source of MVs, how MVs are produced, and with what cells they interact, but this is beyond the scope of the current study. Finally, we have not been able to assess the functional significance of TACE packaged within MVs. The extracellular release of TACE has been reported by Jiang *et al*. (60), and its presence within exosomes has been published by Barberà-Cremades *et al*. (61). However, the presence of TACE within MVs has not been previously reported.

In summary, our results demonstrate that the danger signal ATP critically modulates cytokine secretion from macrophages, redirecting intracellular TNF trafficking and inducing a novel unconventional form of membrane TNF signaling *via* MVs without cell-to-cell contact. These data have important, previously unappreciated biologic implications for this key proinflammatory cytokine, particularly when targeting TNF in drug applications.

## Supplementary Material

This article includes supplemental data. Please visit *http://www.fasebj.org* to obtain this information.

## Abbreviations

ARDS: acute respiratory distress syndrome
ASM: acid sphingomyelinase
BALF: bronchoalveolar lavage fluid
BB94: batimastat
BFA: brefeldin A
BMDM: bone marrow–derived macrophage
CXCL1: chemokine (C-X-C motif) ligand 1
EM: electron microscopy
ER: endoplasmic reticulum
ICAM1: intercellular adhesion molecule 1
IQR: interquartile range
MV: microvesicle
siRNA: small interfering RNA
TACE: TNF-α converting enzyme
WT: wild type
