# Supplementary material for: ATP redirects cytokine trafficking and promotes novel membrane TNF signaling via microvesicles
Source: FASEB J. 2019 Feb 18;33(5):6442–55. doi: 10.1096/fj.201802386R (PMC6463909; doi:10.1096/fj.201802386R)
Supplement: Supplementary file 1 [file fj.201802386R.sf1.pdf]

Supplementary Figure 1. ‘Two-hit’ model in vitro.

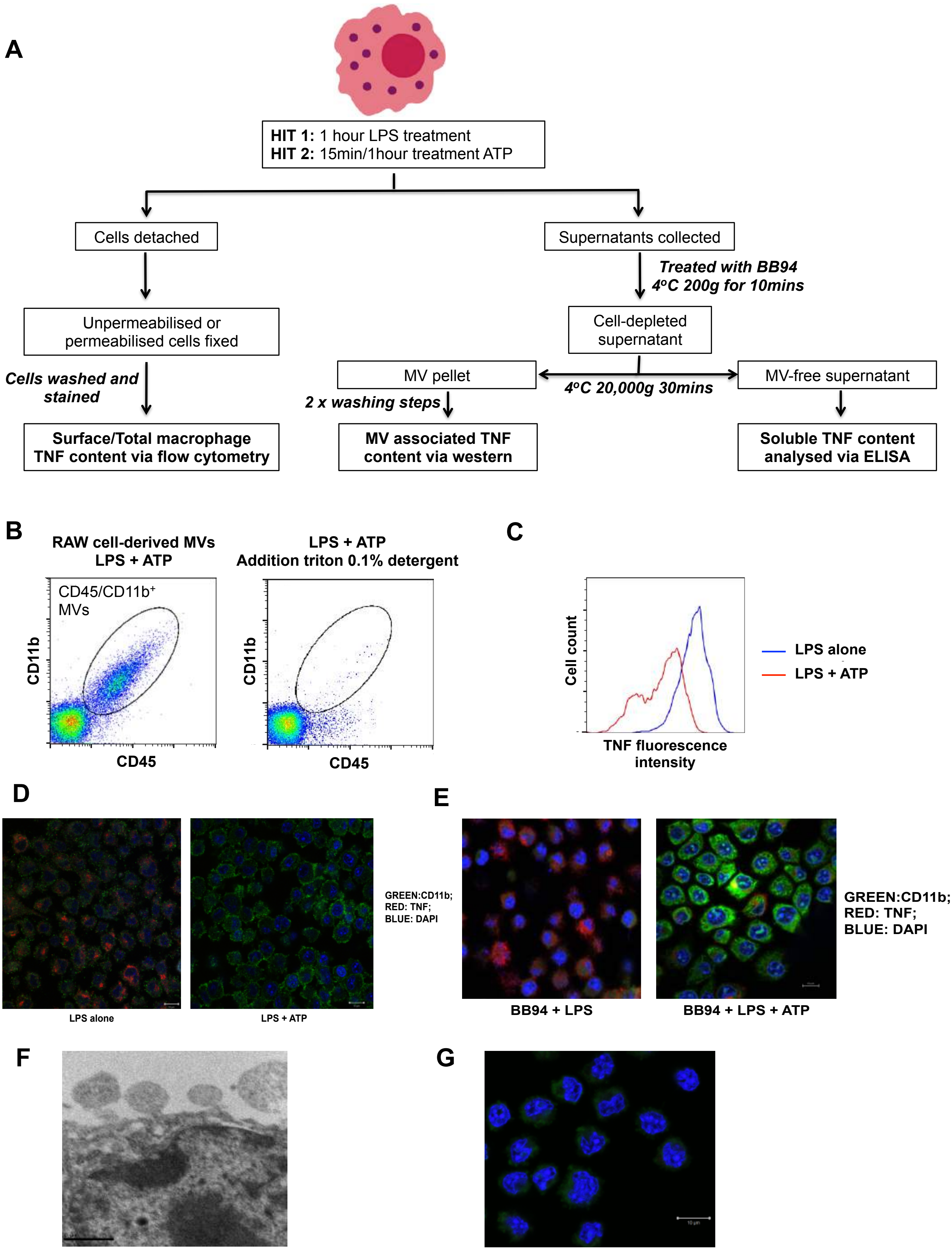

Figure S1: 'Two-hit' model in vitro. (A) 'Two-hit' model to induce production of pro-inflammatory MVs from macrophages in vitro. RAW 264.7 macrophages or bone marrow derived macrophages (BMDMs) were firstly primed with 1µg LPS for 1 hour to induce TNF expression within cells. The cells were then exposed to the nucleotide danger signal ATP (3mM), for either 15 or 60 minutes (or LPS alone for a further 15 or 60 minutes for control) to induce release of pro-inflammatory MVs. Following treatment, supernatants were collected and treated with BB94 to prevent any cleavage of pro-TNF to soluble TNF. Supernatants were then centrifuged for 10mins at 200g 4°C to remove cells/debris and MVs were isolated from cell-free supernatants by high-speed centrifugation (20,000g for 30mins at 4°C), which were washed twice to remove any contaminating factors. Simultaneously cells were harvested from their wells and fixed. Unpermeabilized fixed cells were either stained for TNF (cell surface TNF expression) or permeabilized and stained with TNF (to assess total cellular TNF expression). MV free supernatant was assessed for soluble TNF content and the MV pellet was analysed via western blotting. (B) MVs were identified as CD45+/CD11b+ particles via flow cytometry, and validated as MVs through their sensitivity to 0.1% Triton X-100 detergent, which solubilises lipid membranes (thus becoming undetectable by flow cytometry). (C) Flow cytometry shows the shift in RAW macrophage TNF fluorescence intensity following ATP treatment, highlighting the reduction in cellular TNF expression (representative plot, n=5-6). (B) Confocal images of RAW cells illustrating the reduction of cellular TNF expression following ATP treatment (n=3). (E) This reduction is more significant when RAW cells are pre-treated with BB94 allowing amplification of cellular TNF which was substantially reduced following ATP treatment (n=3). (F) A wide-field immune-electron microscopy image of RAW cells stimulated with ATP without LPS priming. Without LPS priming, TNF (as demonstrated by black dots) is not present within MVs blebbing from cells (n=3). (G) Confocal images demonstrating that RAW cells treated with PBS (i.e. non-stimulated conditions) do not express TNF.

Supplementary Figure 2. ATP simulates MV production from primary bone marrow derived macrophages.

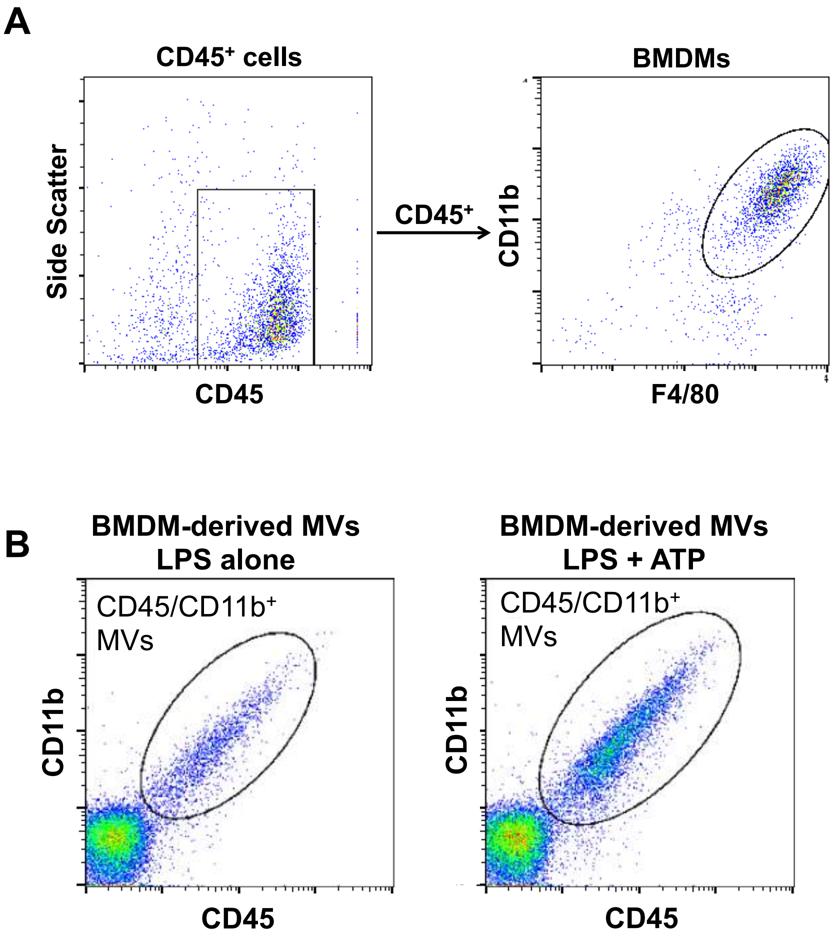

Figure S2: ATP simulates MV production from primary bone marrow derived macrophages. (A) Bone marrow derived macrophages (BMDMs) were identified after differentiation via flow cytometry as $CD45^+$, $F4/80^+$ and $CD11b^+$ cells. (B) BMDM-derived MVs were identified via flow cytometry as $CD45^+/CD11b^+$ particles.

Supplementary Figure 3. In vivo two-hit model.

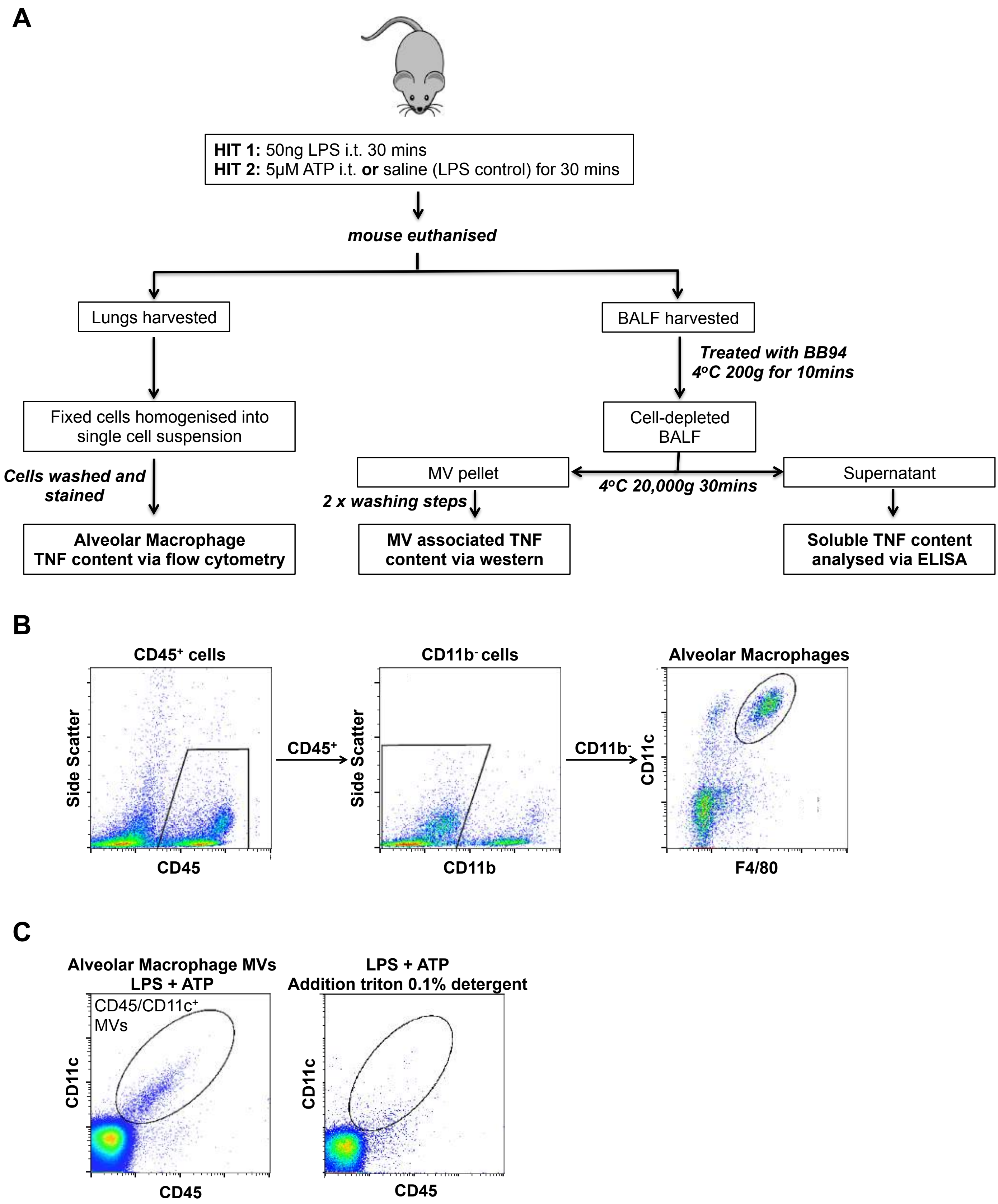

Figure S3: In vivo two-hit model. (A) Protocol to simulate two-hit model in vivo. Low dose LPS (50ng, in 25μl saline) was instilled into the lungs of mice to induce inflammation for 30 minutes. Thereafter a second hit of either 5mM ATP (5μM, in 25μl saline) or saline (termed LPS control) was instilled for a further 30 minutes. After treatment, mice were euthanized, and bronchoalveolar lavage (BALF) was taken (spiked with BB94 to prevent any cleavage of pro-TNF to soluble TNF). BALF was then centrifuged for 10mins at 200g $4^{o}C$ to remove cells/debris and MVs were isolated from cell-free BALF by high-speed centrifugation (20,000g for 30mins at $4^{o}C$), which were washed twice to remove any contaminating factors. MV free supernatant was assessed for soluble TNF content and the MV pellet was analysed via western blotting. Simultaneously, lungs were harvested, dissociated into a single cell suspension, fixed, permeabilized and stained for TNF expression analysis via flow cytometry. (B) Alveolar macrophages were identified in vivo via flow cytometry as $CD45^{+}$, $CD11b^{-}$, $F4/80^{+}$ and $CD11c^{+}$. (C) Alveolar macrophage-derived MVs were identified in vivo via flow cytometry as $CD45^{+}/CD11c^{+}$ particles, which were detergent sensitive.

Supplementary Figure 4. MVs from ASM treated cells.

A

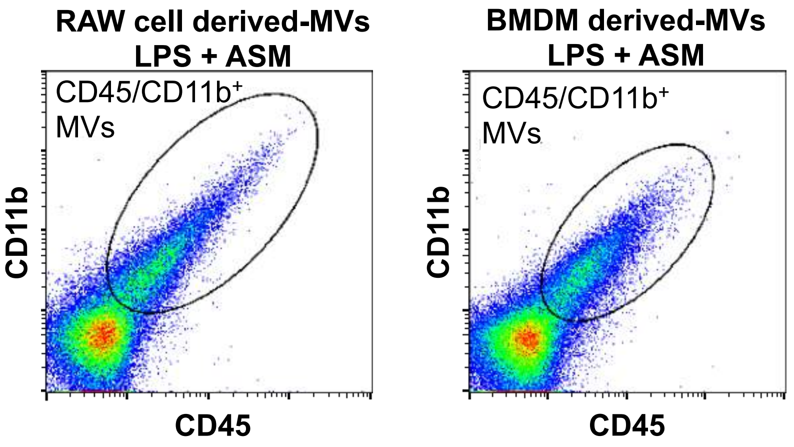

B

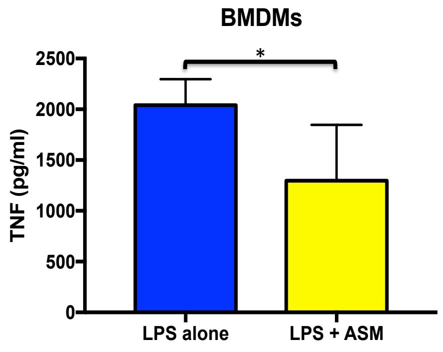

C

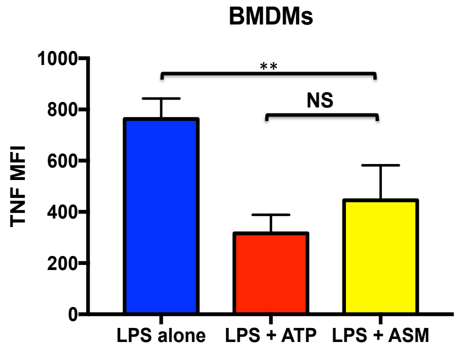

Figure S4: MVs from ASM treated cells. (A) Acid sphingomyelinase (ASM) produces MVs from RAW cell (left) and BMDMs (right) as depicted by the $CD45^+/CD11b^+$ in the flow cytometry plots. (B) Like ATP, recombinant ASM inhibits soluble TNF release (n=4). (C) ASM concurrently reduces total macrophage TNF content to a level comparable with ATP (right) (n=4). Parametric data displayed as mean ± s.d. showing the median, IQR and minimum/maximum values respectively. *p<0.05, **p<0.01, ***p<0.001

Supplementary Figure 5. MVs cause inflammation in vivo via a TNF dependent manner.

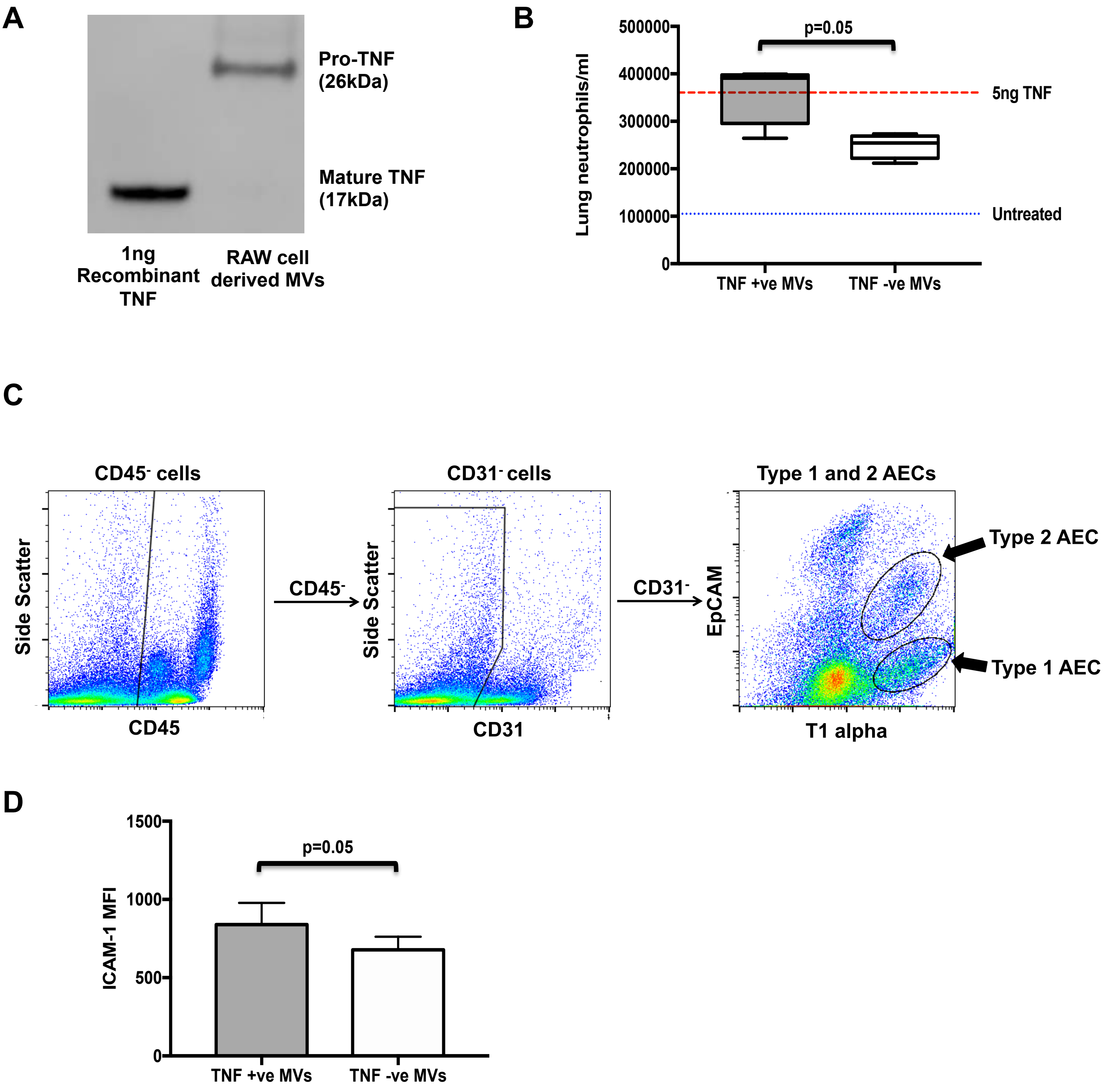

Figure S5: MVs cause inflammation in vivo via a TNF dependent manner. (A) Comparison of 1ng recombinant TNF versus the pro-TNF content within RAW cell derived MVs. (B) BMDM MVs obtained from wild type mice were instilled i.t. into the lung of mice causing an increase in lung neutrophil infiltration compared to MVs obtained from TNF knockout BMDMs although this did not quite reach statistical significance (n=4). (C) Alveolar epithelial cells (AECs) were identified via flow cytometry as $CD45^-$, $CD31^-$, $T1alpha^+$ and $EpCAM^-$ (type 1 AECs) and $T1alpha^+$ and $EpCAM^+$ (type 2 AECs). (D) BMDM MVs from wild type mice caused an increase in ICAM-1 expression on type 2 AECs compared to MVs obtained from TNF knockout BMDMs although this did not quite reach statistical significance (n=4). Parametric/non parametric data displayed as mean ± s.d. or box–whisker plots showing the median, IQR and minimum/maximum values respectively.
